# Spina Bifida and primary prevention

**DOI:** 10.1186/1750-1172-7-S2-A18

**Published:** 2012-11-22

**Authors:** Pierre Mertens

**Affiliations:** 1 International Federation for Spina Bifida and Hydrocephalus (IF), Brussels, Belgium

## 

Spina Bifida is a neural tube defect (NTD), which occurs within the first 25 days of pregnancy and affects around 1 in 1000 pregnancies in Europe. It cannot be cured, although improved medical interventions mean that many people with Spina Bifida live into old age and have a good quality of life. However lifelong follow-up is required. IF is an umbrella organisation of national organisations of persons with Spina Bifida and focuses on primary prevention, access to health and the right to life. Around 70% of Spina Bifida can be prevented by a daily intake of Folic Acid two months prior to conception and two after. With Folic Acid the same child is born without Spina Bifida. Access to life-saving treatment of new-borns with Spina Bifida is in discussion. IF strives for the right of treatment of all new-borns with Spina Bifida. Spina Bifida became a rare disease because of prenatal detection and terminations of pregnancies and not because of primary prevention. Several hospitals closed their coordinated care units for Spina Bifida. Research decreased and Spina Bifida is perceived by many professionals as a solved problem. Prevention of most of the birth defects starts before conception. That's why IF co-organised in October 2010 a European Preconception Care conference in Brussels. When a couple plans their pregnancy they should take all measures to prevent possible problems: not drinking alcohol, not smoking, not taking drugs and taking Folic Acid. IF advocates for fortification of staple food with Folic Acid and set up a European awareness campaign. Most women do not know about the risk of having a baby affected by an NTDs. This is even truer among women of lower socio-economic status where the incidence is higher. IF produced two reports on Prevention of NTD's in Europe and organised a hearing in the European Parliament. To improve peri-conceptional folate levels women of childbearing age can only be reached by a combination of counselling, intake of Folic Acid, fortified staple food and further investment in monitoring and research. Persons with Spina Bifida make the need for prevention visible and IF believes that it should be integrated in National Plans for rare diseases. In Belgium we had a first success. A crucial orphan drug in the care for the neurogenic bladder is taken for approval.

